# Adipose tissue-derived omentin-1 attenuates arterial calcification via AMPK/Akt signaling pathway

**DOI:** 10.18632/aging.102251

**Published:** 2019-10-25

**Authors:** Feng Xu, Fu-Xing-Zi Li, Xiao Lin, Jia-Yu Zhong, Feng Wu, Su-Kang Shan, Chang-Ming Tan, Ling-Qing Yuan, Xiao-Bo Liao

**Affiliations:** 1 Department of Endocrinology and Metabolism, Hunan Provincial Key Laboratory of Metabolic Bone Diseases, National Clinical Research Center for Metabolic Diseases, The Second Xiang-Ya Hospital, Central South University, Changsha 410011, Hunan, People’s Republic of China; 2 Department of Geriatrics, The Second Xiang-Ya Hospital, Central South University, Changsha 410011, People’s Republic of China; 3 Department of Pathology, The Second Xiang-Ya Hospital, Central South University, Changsha 410011, People’s Republic of China; 4 Department of Cardiovascular Surgery, The Second Xiang-Ya Hospital, Central South University, Changsha 410011, People’s Republic of China

**Keywords:** Akt, AMP-activated protein kinase, omentin-1, smooth muscle differentiation, arterial calcification

## Abstract

Adipose tissue-derived adipokines mediate various kind of crosstalk between adipose tissue and other organs and thus regulate metabolism balance, inflammation state as well as disease progression. In particular, omentin-1, a newly found adipokine, has been reported to exhibit anti-calcification effects *in vitro* and *in vivo*. However, little is known about the function of endogenous adipose tissue-derived omentin-1 in arterial calcification and the detailed mechanism involved. Here, we demonstrated that global omentin-1 knockout (omentin-1^-/-^) resulted in more obvious arterial calcification in 5/6-nephrectomy plus high phosphate diet treated (5/6 NTP) mice while overexpression of omentin-1 attenuated attenuates osteoblastic differentiation and mineralisation of VSMCs *in vitro* and 5/6 NTP-induced mice arterial calcification *in vivo*. Moreover, we found that omentin-1 induced AMPK and Akt activation while inhibition of AMP-activated protein kinase (AMPK) and Akt signaling reversed the anti-calcification effect induced by omentin-1 both *in vitro* and *in vivo*. Our results suggest that adipose tissue-derived omentin-1 serves as a potential therapeutic target for arterial calcification and cardiovascular disease.

## INTRODUCTION

With the growing tendency of global aging, age-associated cardiovascular disease has emerged as a serious global burden upon human health and economic development [1, 2]. Among them, vascular calcification has gradually gained much attention as it relates to higher risk of cardiovascular events [3] and serves as a marker of the aged artery with progressively phenotype transition of contractile VSMCs into “synthetic” VSMCs [4]. With the advent of deep research into this area, vascular calcification has now been demonstrated to be an active systematic process sharing many similarities with mineralisation of skeleton [5, 6]. This process contains the “ectopic” expression of some osteoblastic phenotype genes such as alkaline phosphatase (ALP), runt related transcription factor 2 (Runx2), osteocalcin (OC) and osteopontin, and involves mineral matrix microenvironment [7]. Moreover, several studies have identified that osteogenic conversion of VSMCs plays a predominant role in arterial calcification [8, 9].

Accumulating evidence has demonstrated that fat tissue serves as more than a fat storage station; it also functions as a novel huge “endocrine organ,” secreting abundant adipokines such as leptin and adiponectin, among others, which link complex network between adipose tissues and cardiovascular system and some others [10–12]. Omentin-1, also referred to as intelectin-1, is a novel adipocytokine that exists abundantly in human visceral fat tissue and has been reported to be detectable in human plasma [13]. The circulation level of omentin-1 is reduced in obesity and obesity-related diseases such as impaired glucose regulation and type-2 diabetes [14, 15]. Recent advances have demonstrated that omentin-1 inhibits tumor necrosis factor-induced vascular inflammation and stimulates endothelial cell function [16, 17]. However, the role of endogenous omentin-1 in mediating adipose-cardiovascular system interplay is just beginning to be determined.

AMPK cascade is best known as a sensor of cellular energy status, which further regulates whole-body energy balance, particularly by transmitting the function of hormones and cytokines such as leptin, adiponectin and ghrelin [18]. Some studies reveal that omentin-1 induces AMPK phosphorylation, thus preventing myocardial ischemic injury and attenuating cardiac hypertrophic response [19, 20]. Recent evidence shows that AMPK signaling pathway was involved in osteogenic differentiation of VSMCs [21]. In addition, accumulating evidence verifies that Akt, also known as protein kinase B (PKB), lies downstream of AMPK signaling pathway and mediates various physiologic and pathological cell processes and multiple diseases progression [22, 23].

Some recent studies have reported that omentin-1 regulates vascular calcification. For example, Duan et al [24] reported that omentin-1 inhibits osteoblastic differentiation of calcifying vascular smooth muscle cells through the PI3K/Akt pathway *in vitro*. This effect was inconsistent with *in vivo* study that overexpression of omentin-1 could ameliorate arterial calcification via inhibition of Receptor Activator of Nuclear Factor-κB Ligand (RANKL) production in osteoprotegerin knockout mice [25]. However, no study has explored the effects of genetic deletion of omentin-1 on vascular functions. Here, we demonstrate that adipose tissue-derived endogenous omentin-1 protects against arterial calcification. Moreover, AMPK/Akt mediates the anti-calcification effects of omentin-1 *in vitro* and *in vivo*. These results give rise to the advance of research into adipose-cardiovascular interaction and shed new light on adipose tissue as a potential target of cardiovascular disease therapy.

## RESULTS

### Omentin-1 attenuates osteoblastic differentiation and mineralisation of VSMCs

To identify the effect of omentin-1 on osteoblastic differentiation of mouse calcified vascular smooth muscle cells (CVSMCs) and β-GP-treated primary human arterial VSMCs (HAVSMCs), two classical cell models of osteoblastic differentiation of VSMCs, recombinant human omentin-1 was added to culture medium of both kinds of cells at concentrations ranging from 100 to 400 ng/ml.

In this study, the activity of ALP, the secretion of OC and the expression of Runx2 were measured to identify the function of omentin-1 on phenotype transition of VSMCs. Calcium deposition detection and Alizarin Red S staining were performed to evaluate the mineralisation level of VSMCs.

**Figure 1:**
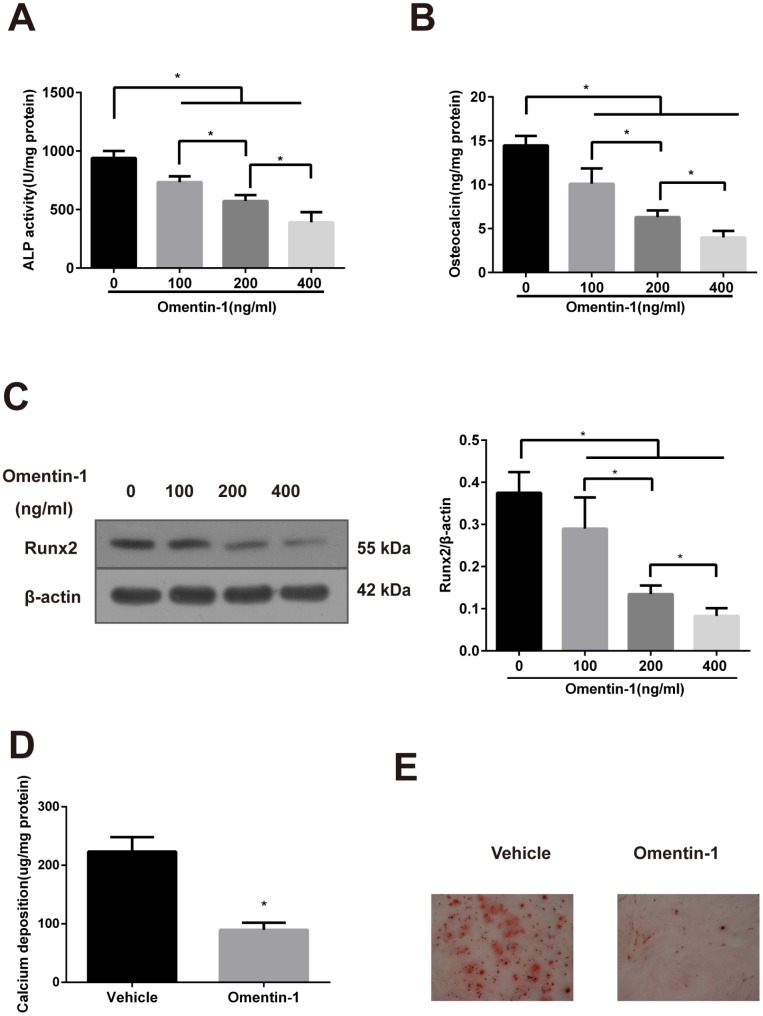
**Effects of omentin-1 on osteoblastic differentiation and mineralisation of calcifying vascular smooth muscle cells (CVSMCs).** (**A**) Effects of omentin-1 on the ALP activity in CVSMCs. The cells were cultured with or without omentin-1 (100-400 ng/mL) for the indicated time points. The ALP activity was measured by an ALP kit, normalized to the cellular protein contents. *p < 0.05, one-way ANOVA with the Tukey’s HSD post hoc analysis. (**B**) Effects of omentin-1 on osteocalcin (OC) secretion in CVSMCs. The cells were treated with or without omentin-1 (100–400 ng/mL) for 48 hours, and then OC secretion of the cells was determined by radioimmunoassay, normalized to the cellular protein contents. *p < 0.05, one-way ANOVA with the Tukey’s HSD post hoc analysis. (**C**) Effects of omentin-1 on Runx2 expression in CVSMCs. The cells were cultured for 48 hours with or without 100-400 ng/mL omentin-1. The data were presented as densitometric ratios of Runx2/β –actin. *p < 0.05, one-way ANOVA with the Tukey’s HSD post hoc analysis. (**D**) Effects of omentin-1 on calcium deposition. The cells were cultured for 14 days with or without omentin-1. *p < 0.05, unpaired Student’s t-test. (**E**) Alizarin Red S staining view of either vehicle or omentin-1 treated CVSMCs for 14 days. Representative microscopic pictures were shown. All Results are represented by mean ± SD with 3 replicates for each group.

CVSMCs are a universal calcifying model that can spontaneously express the osteoblast phenotype genes and form calcification nodules [26]. As shown in Figure 1A, the ALP activity in CVSMCs was suppressed by omentin-1 in a dose-dependent manner. Moreover, the inhibition effects of OC secretion and expression of Runx2 protein of omentin-1 in a dose-dependent manner were also observed (Figure 1B, 1C). Furthermore, the effects of omentin-1 on calcium deposition and matrix calcification in CVSMCs were examined after 14-day incubation. The results showed that omentin-1 significantly decreased matrix calcification and calcium deposition (Figure 1D, 1E). Taken together, these findings demonstrate that omentin-1 attenuates CVSMCs osteoblastic differentiation and mineralisation.

To further identify omentin-1 induced inhibition effects on VSMCs calcification, we isolated HAVSMCs and then treated HAVSMCs with recombinant omentin-1 and β-GP. Consistent with our previous results, increasing concentrations of omentin-1 treatment significantly inhibited β-GP-induced osteogenic conversion of HAVSMCs, as demonstrated by markedly decreased the ALP activity (Supplementary Figure 1A), the OC secretion (Supplementary Figure 1B) and the expression of Runx2 protein (Supplementary Figure 1C) in a dose-dependent manner. Moreover, matrix mineralisation in HAVSMCs cultured with indicated medium for 21-day incubation was evaluated by detecting calcium deposition and Alizarin Red S staining. The results showed that omentin-1 treatment remarkably reduced the calcium content and Alizarin Red S staining level of β-GP treated HAVSMCs (Supplementary Figure 1D and 1E).

These data together demonstrate that omentin-1 inhibits osteogenic differentiation and mineralisation in both β-GP-induced VSMCs and CVSMCs.

### Deletion of omentin-1 accelerates 5/6 NTP-induced arterial calcification in mice

**Figure 2:**
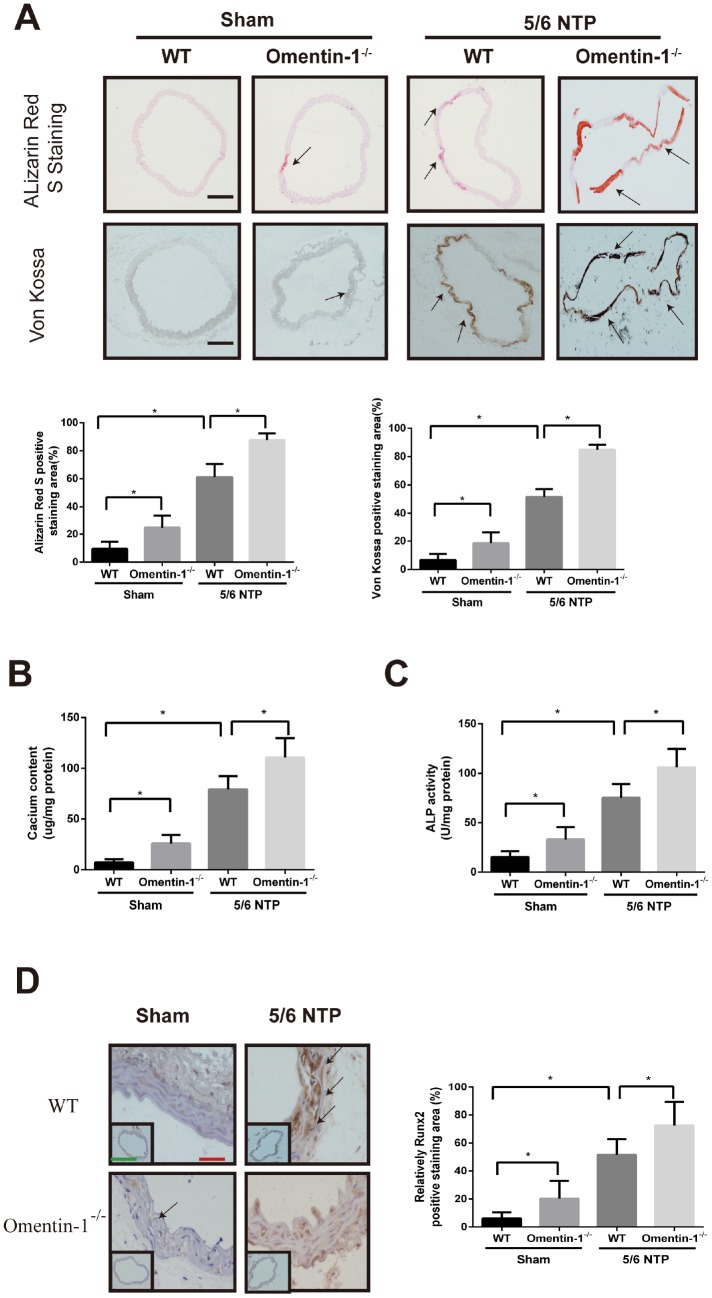
**Omentin-1 deficient enhanced artery calcification in 5/6 NTP-induced mice.** Six-week-old omentin-1-/- mice and their littermates were subjected to 5/6-nephrectomy or sham operation following by high phosphate diet (0.9% Pi) for indicated time. (**A**) Sections from the thoracic aorta of sham operation mice and 5/6 NTP mice were determined by Alizarin Red S staining and Von Kossa staining. Representative microscopic pictures were shown (upper panel) and quantity of positive staining area in the thoracic aorta were analyzed (lower panel). Scale bar 200 μm (n=6/group). (**B**) Calcium content of the thoracic aorta was measured in different group mice. (**C**). The ALP activity of the thoracic aorta was measured by an ALP kit, normalized to the total tissue protein contents. (**D**) Immunohistochemistry of Runx2 in the mouse thoracic aorta. Representative microscopic pictures were shown in the upper panel and quantity of positive staining area in the thoracic aorta were shown in the lower panel. Scale bar 20 μm (Red) and 500 μm (Green). Results are represented by mean ± SD with 6 replicates for each group. Significance was analyzed by two-way ANOVA with the Tukey’s HSD post hoc analysis. (*p < 0.05).

To investigate whether omentin-1 plays a role in the process of endogenous arterial calcification progression, we constructed global omentin-1 null mice and analyzed calcification level by using 5/6-nephrectomy (5/6 NTP) induced mice as *in vivo* calcification model. Briefly, omentin-1^-/-^ mice and their wild-type littermates (WT) were subjected to 5/6 nephrectomy or sham operation following by high phosphate diet treatment and the level of arterial calcification was detected. Deletion of omentin-1 was confirmed by the absence of protein expression in adipose tissue and undetectable level in plasma of omentin-1^-/-^ mice (Supplementary Figure 2A, 2B). Meanwhile, circulating plasma omentin-1 level was decreased in 5/6 NTP mice when compared with control mice (Supplementary Figure 2B). No obvious Von Kossa and Alizarin Red S staining was found in WT mice after sham operation (Figure 2A). Intriguingly, the arterial calcification level of sham control omentin-1^-/-^ mice ranged from undetectable to just slight. In contrast, significant artery calcification was observed in NTP-induced mice and more obvious staining was found in omentin-1^-/-^ mice compared with their wild-type littermates (Figure 2A). Moreover, our results demonstrated that aortic calcium content, ALP activities and Runx2 immunostaining were increased in NTP-induced omentin-1^-/-^ mice when compared with their wild-type littermates and sham control omentin-1^-/-^ mice (Figure 2B–2D). These results show that the omentin-1 serves as a protective factor in NTP-induced calcification in mice.

### Omentin-1 activates AMPK and Akt signaling in CVSMCs and 5/6 nephrectomy mice

**Figure 3:**
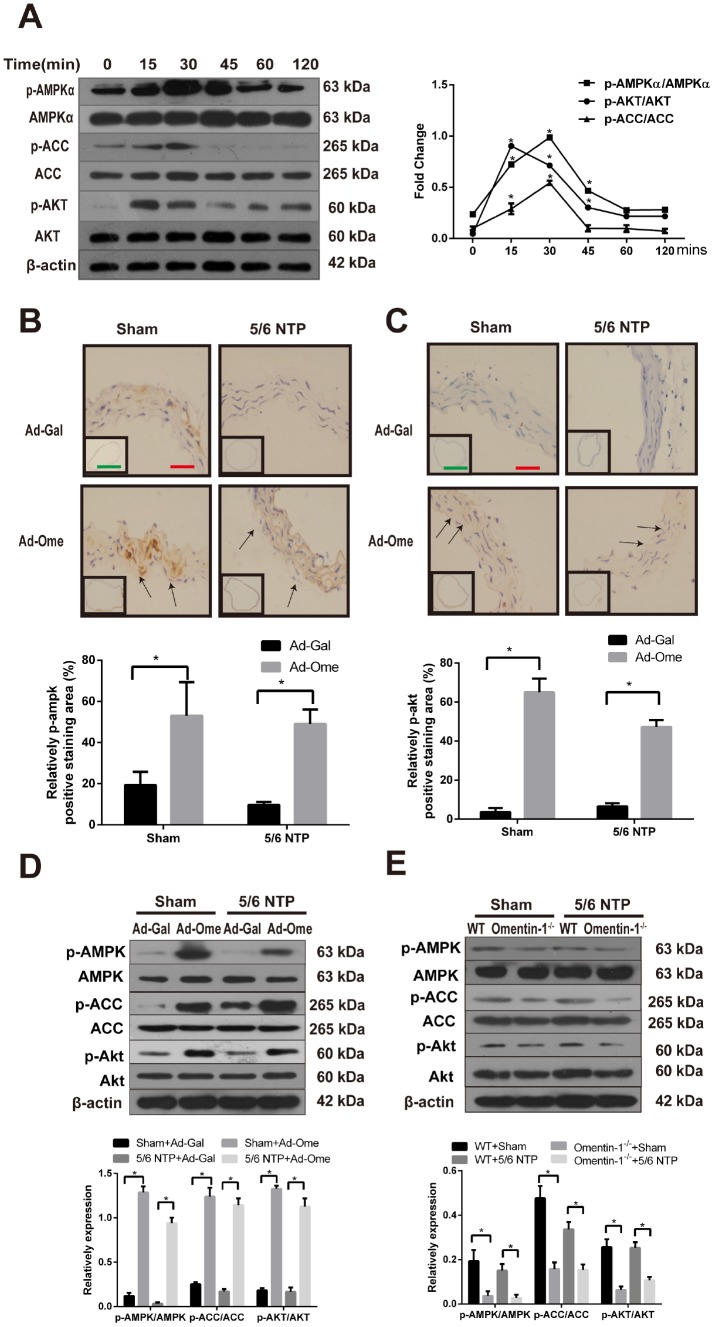
**Effects of omentin-1 on activation of AMPK and Akt phosphorylation in CVSMCs and the 5/6 NTP-induced mouse calcification model.** (**A**) CVSMCs were incubated with 400 ng/ml omentin-1 for either 0, 15, 30, 45, 60, or 120 min. Cells lysates were tested by western blot and incubated with antibodies against p-AMPKα, AMPKα, p-Akt, Akt, p-ACC and ACC. Representative results were shown in the left panel and densitometric quantification analysis for phosphorylation of Akt, AMPK and ACC was presented in the right panel. *p < 0.05 vs. respective 0 min, n=3, one-way ANOVA with the Tukey’s HSD post hoc analysis. Mice were subjected to 5/6-nephrectomya or sham operation following by high phosphate diet (0.9% Pi) for indicated time and then the adenovirus-encoding omentin-1 (Ad-Ome) or control adenovirus-encoding β-galactosidase (Ad-Gal) were injected into the tail vein of mice one time per week for four weeks. Expression of p-AMPKα and p-Akt were analyzed by immunohistochemistry. (**B**) Representative immunostaining of phosphorylation of AMPK proteins (upper panel) and quantity of positive staining area in the thoracic aorta were shown in the lower panel (lower panel). (**C**) Representative immunostaining of phosphorylation of Akt proteins (upper panel) and quantity of positive staining area in the thoracic aorta were shown in the lower panel (lower panel). (**D**) Expression of p-AMPK, p-ACC and p-Akt in the mouse aorta treated with Ad-Ome or Ad-Gal were analyzed by western blot. (**E**) Expression of p-AMPK, p-ACC and p-Akt in the aorta of omentin-1 knockout mice or wild type mice were analyzed by western blot. Scale bar 20 μm (Red) and 500 μm (Green). *p < 0.05 vs. respective control group, n=6, two-way ANOVA with the Tukey’s HSD post hoc analysis. All Results are represented by mean ± SD.

The AMPK is known as a sensor of cellular energy and nutrient status and recent studies have demonstrated that AMPK is involved in osteoblastic differentiation [21, 27]. Previous work also shown that Akt signaling pathway was activated after treatment of omentin [24]. In this study, we further investigate the role of AMPK in omentin-1 induced inhibition effects on osteogenic conversion of VSMCs and the crosstalk between AMPK and Akt in this process. CVSMCs were treated with omentin-1 (400ng/ml) and the phosphorylation level of AMPKα, acetyl-CoA carboxylase (ACC) and Akt were examined by western blot. Phosphorylation of AMPK was firstly observed at 15 minutes and reached the highest point at 30 minutes after induction of omentin-1 in CVSMCs, while no significant change was observed in total AMPK level in the same period (Figure 3A). Moreover, phosphorylation of ACC, one of the most important downstream targets of AMPK, was also induced by omentin-1 treatment (Figure 3A). Meanwhile, we found marked activation of Akt after omentin-1 treatment for 15 min. Next, we detected phosphorylation of AMPK and Akt level in NTP-induced mice after tail vein injection of Ad-Ome. Similarly, we found that overexpression of omentin-1 significantly increased the immunostaining level of AMPK, Akt in the mouse aorta (Figure 3B, 3C). Intriguingly, western blot analysis showed that overexpression of omentin-1 also stimulated AMPK, ACC and Akt phosphorylation in mice (Figure 3D). However, deficient of omentin-1 resulted in decreased AMPK, ACC and Akt phosphorylation level in the aorta of both sham and 5/6NTP operation mice when compared with that of WT mice (Figure 3E). Taken together, these results suggest that AMPK and Akt signaling pathway may play a key role in omentin-1-induced inhibition of VSMC calcification and artery calcification.

### AMPK/Akt signaling pathway mediates inhibition effects of omentin-1 on osteoblastic differentiation and mineralisation of VSMCs

**Figure 4:**
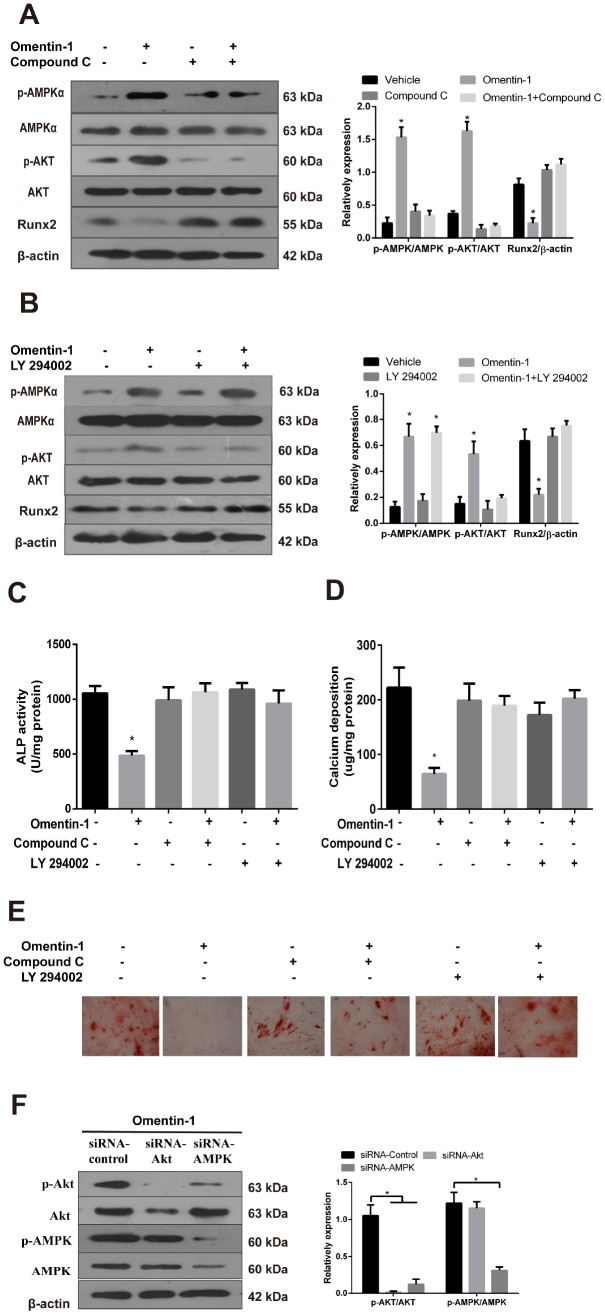
**The AMPK/Akt signaling pathway mediates omentin-1-induced inhibition effects on osteoblastic differentiation and mineralisation of CVSMCs.** CVSMCs were stimulated by Compound C (10 μM) or LY 294002 (30 μM) for 30 min and then treated with omentin-1 (400 ng/ml) for 48 hours. The cell lysates were tested by western blot and incubated with antibodies against p-AMPKα, AMPKα, p-Akt, Akt and Runx2. (**A**, **B**) Representative results were shown in the left panel and the data were presented as densitometric ratios of p-AMPK/AMPK, p-Akt/Akt and Runx2/β–actin respectively (lower panel). (**C**) The ALP activity was measured by ALP kit. Results are represented by mean ± SD with 3 replicates for each group. (*p < 0.05). One-way ANOVA with the Tukey’s HSD post hoc analysis was adopted. (**D**, **E**) CVSMCs were treated with Compound C (10 μM) or LY 294002 (30 μM) for 30 min and then treated with omentin-1 (400 ng/ml) every two days for a period of 14 days. Calcium deposition was tested (**D**) and represented microscopic pictures of Alizarin Red S staining view were shown (**E**). (**F**) Expression of p-AMPK and p-Akt in CVSMCs after AMPK or Akt knockdown were analyzed by western blot. All Results are represented by mean ± SD with 3 replicates for each group. One-way ANOVA with the Tukey’s HSD post hoc analysis was adopted.

To further confirm the involvement of AMPK and Akt activation in omentin-1 induced inhibition of VSMC calcification, CVSMCs were treated with or without omentin-1 (400 ng/ml), or Compound C (10 μM), an inhibitor of AMPK, or LY 294002, an inhibitor of Akt. In the presence of Compound C, omentin-1 induced inhibition effects on the Runx2 protein expression (Figure 4A), the ALP activity (Figure 4C), calcium deposition (Figure 4D) and Alizarin Red S staining level (Figure 4E) were abolished. Similarly, we also found that inhibition of Akt mimicked the effects of Compound C (Figure 4B–4E). Interestingly, inhibition of Akt had no effect on the omentin-1 induced phosphorylation of AMPK (Figure 4B), while inhibition of AMPK significantly abolished omentin-1 induced phosphorylation of Akt (Figure 4A). To further examine the relationship between PI3K and AMPK, we knocked down AMPK and Akt by specific siRNA and then observed changes in p-AMPK and p-Akt. The results showed that AMPK knockdown blocked omentin-1 induced AMPK and Akt phosphorylation, while Akt knockdown only blocked omentin-1 induced Akt activation but not AMPK activation (Figure 4F), indicating that Akt is downstream of AMPK in omentin-1 induced signaling activation. Thus, these experiments demonstrate that omentin-1 inhibits osteoblastic differentiation and mineralisation of VSMCs dependent on AMPK/Akt signaling pathway.

### Overexpression of omentin-1 reduces NTP-induced artery calcification via AMPK/Akt signaling pathway in mice

**Figure 5:**
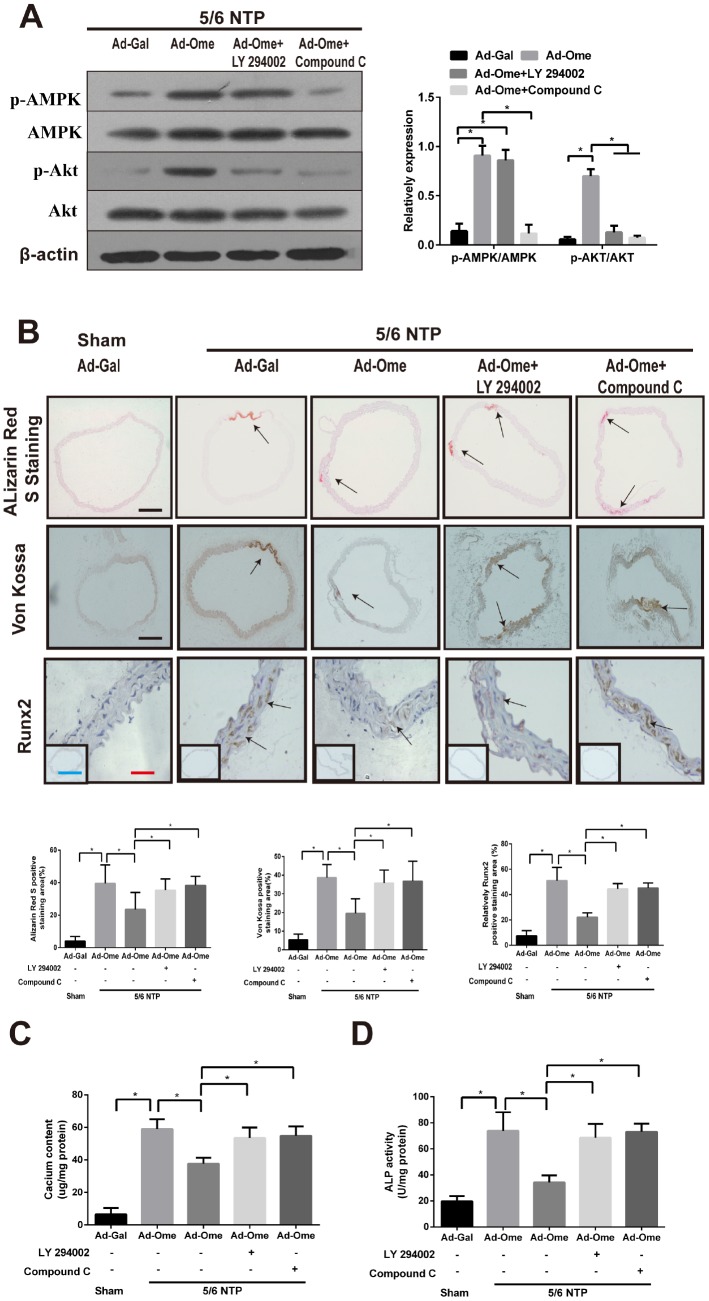
**Omentin-1 alleviates arterial calcification in 5/6 NTP-induced mice.** Six-week-old Male mice were subjected to 5/6-nephrectomy or sham operation following by high phosphate diet (0.9% Pi) for indicated time and then the adenovirus-encoding omentin-1 (Ad-Ome) or control adenovirus-encoding β-galactosidase (Ad-Gal) were injected into the tail vein of mice one time per week for four weeks. (n=6 for each group). (**A**) Expression of p-AMPK and p-Akt in the aorta of 5/6NTP mice treated with LY 294002 or Compound C were analyzed by western blot. (**B**) Von Kossa-stained, Alizarin Red S-stained and Runx2-stained sections from the thoracic aorta were tested to identify calcification level of artery. Representative microscopic pictures were shown in the upper panel and quantity of positive staining area in the thoracic aorta was shown in the lower panel. Scare bar 20 μm (Red), 200 μm (Black) and 500 μm (Green). (**C**) Calcium content of the thoracic aorta was measured in different group mice. (**D**) The ALP activity was measured by an ALP kit, normalized to the total tissue protein contents. Results are represented by mean ± SD with 6 replicates for each group. Significance was analyzed by two-way ANOVA with the Tukey’s HSD post hoc analysis. (*p < 0.05).

To elucidate the effect of omentin-1 on vascular calcification *in vivo*, we performed 5/6 nephrectomy in C57BL/6 mice with sham operation as control mice. All these mice were raised with high–phosphate diet for twelve weeks following by tail vein injection of Ad-Ome or Ad-Gal as a negative control for four weeks. Alizarin Red S staining and Von Kossa staining were used to determine artery calcification in the thoracic aorta tissue. Calcium content and the ALP activity of the thoracic aorta tissue were also determined. Moreover, the thoracic aorta Runx2 expression was tested by immunochemistry. Phosphorylation of AMPK and Akt in the aorta after treatment of PI3K inhibitor or Compound were confirmed by western blot. Increased circulating human omentin-1 after four times of Ad-Ome administration were confirmed as shown in Supplementary Table 1. The results shown that treatment of PI3K inhibitor LY294002 blocked Ad-Ome-induced Akt phosphorylation but not AMPK phosphorylation in the aorta of 5/6 NTP mice, while AMPK inhibitor Compound C blocked both Akt and AMPK phosphorylation induced by Ad-Ome (Figure 5A). 5/6 NTP plus high-phosphate diet significantly increased Alizarin Red S staining level, Von Kossa staining level (Figure 5B), calcium content (Figure 5C) and the ALP activity (Figure 5D) in the thoracic tissues compared with those of sham control group. In consistent, Runx2 expression was increased in 5/6 NTP mice compared with sham control mice, further demonstrating that 5/6 NTP mice successfully developed artery calcification (Figure 5B). However, overexpression of omentin-1 partially alleviated 5/6 NTP-induced arterial calcification compared with Ad-Gal control group, as demonstrated by reduced immunostaining of Von Kossa, Alizarin Red S, Runx2 (Figure 5B), decreased level of calcium content and the ALP activity (Figure 5C, 5D) in the thoracic aorta tissues. Furthermore, Pretreatment with either Akt or AMPK inhibitors abolished omentin-1 induced anti-calcification effects (Figure 5B–5D). These results strongly suggest that omentin-1 acts through activation of AMPK/Akt signaling pathway to exert anti-calcification effects in 5/6 NTP induced mice.

## DISCUSSION

**Figure 6:**
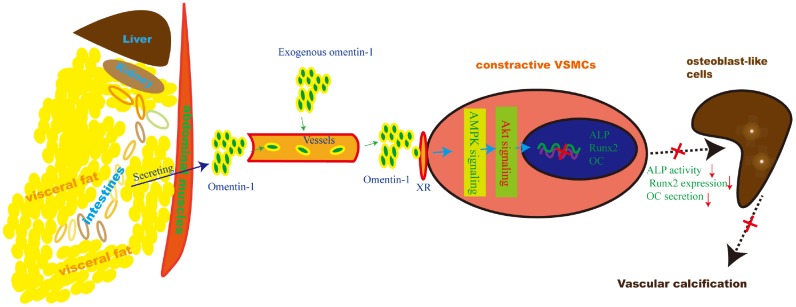
**Schematic model of the omentin-1 induced anti-calcification effect.** In this study, we demonstrate that omentin-1, mainly derived from visceral fat tissues, induces the AMPK/Akt signaling activation in VSMCs and thus attenuates the expression of Runx2, reduces ALP activity and decreases OC secretion. As a result, arterial calcification is alleviated.

Herein, we provide reliable evidence that omentin-1 mediates adipose-cardiovascular system interaction. Supplementation of exogenous human omentin-1 reduced VSMCs calcification *in vitro* and alleviated 5/6 NTP-induced arterial calcification *in vivo* while deletion of endogenous omentin-1 accelerated arterial calcification in 5/6 NTP-induced mice. Moreover, Inhibition of both AMPK and Akt abolished omentin-1 induced anti-calcification effects in calcifying VSMCs and 5/6 NTP-induced arterial calcification in mice. These data suggest that adipose-derived omentin-1 activates AMPK/Akt signaling in the aorta VSMCs and then inhibits osteoblastic differentiation of VSMCs, resulting in reduced arterial calcification (Figure 6). These findings also open up new windows to explore the role of omentin-1 in mediating adipose tissue and other organs or systems interaction.

Much evidence has confirmed that arterial calcification resembles osteogenesis, and arterial calcification is an actively regulated process. During this process, osteoblastic-specific marker genes are significantly increased and smooth muscle lineage markers genes are decreased, resulting in arterial medial calcification. We and others have identified that adipokines regulate osteogenic differentiation, proliferation and apoptosis of VSMCs [26, 28, 29]. More recently, Duan et al [24] investigated the effect and mechanism of omentin-1 in CVSMCs and they found that 500 ng/ml omentin-1 significantly reduced CVSMCs calcification in vitro [24]. In the present study, we further investigated the effect of omentin-1 on arterial calcification both *in vitro* by using both primary human VSMCs and mice CVSMCs. In agreement with previous reports, we found omentin-1 decreased the ALP activity, osteocalcin secretion, the Runx2 expression and mineralized nodules formation in both β-GP-induced HAVSMCs and mice CVSMCs *in vitro* at the dose of 100 ng/ml to 400 ng/ml. We have demonstrated that exogenous overexpression of omentin-1 by systemic delivery of Ad-Ome reduces mice arterial calcification in OPG^-/-^ mice [25]. Actually, progressive arterial calcification observed in OPG^-/-^ mice is owing to loss of the protection factor OPG instead of actual disease states. However, 5/6 NTP-induced mice stimulate ESRD states related arterial calcification that is common found in ESRD patients and thereby serves as the desirable mice model to display arterial calcification [30, 31]. As expected, 5/6 nephrectomy plus high phosphate diet resulted in prominent arterial calcification in the mouse aorta, as compared with the sham control mice. Moreover, we found that overexpression of omentin-1 via tail vein injection of Ad-Ome could markedly reduce the 5/6 NTP-induced arterial calcification in mice. These data demonstrate that omentin-1 attenuates arterial calcification both *in vitro* and *in vivo*.

Emerging evidence shows that exogenous omentin-1 exhibits protection effect on arterial calcification. However, the role of endogenous omentin-1 in the development of arterial calcification is largely unknown. In this study, we constructed omentin-1 knockout mice to identify the protective effect of endogenous omentin-1 on arterial calcification. Interestingly, we found that immunostaining of Von Kossa and Alizarin Red S Staining ranged from absent to slightly detectable in omentin-1^-/-^ mouse aorta along with increased activity of ALP, calcium content as well as Runx2 expression. These observations were in consistent with the phenotype of adiponectin global knockout mice that Alizarin red S staining level was also significantly increased in adiponectin deficient mice [32]. More importantly, we also explore the role of omentin-1 in NTP-induced mouse calcification model. We found that omentin-1^-/-^ mice developed more serious calcification in 5/6 NTP-induced mice when compared with their WT littermates, as demonstrated by significantly increased calcification area and calcium content as well as upregulated calcification marker genes expression. Taken together, these findings indicate that omentin-1 is required for regulation of arterial balance and strongly suggest that endogenous omentin-1 protects against arterial calcification.

It has been demonstrated that both AMPK and Akt signaling pathway plays an important role in cellular differentiation and thus regulates vascular functions [25, 33, 34]. Moreover, several studies have revealed omentin-1 stimulates AMPK or Akt activation in myocyte and endothelial cells as well as ischemic hearts of mice [16, 17, 19]. Consistent with these findings, exogenous omentin-1 induced remarkable activation of AMPK and Akt in CVSMCs and 5/6 NTP induced mice aorta, while omentin-1 induced inhibitory effects on osteoblastic differentiation of CVSMCs and mice arterial calcification were significantly abolished by AMPK inhibitors or Akt inhibitors. We also investigated the relationship between AMPK and Akt signaling in omentin-1 treated CVSMCs in vitro. We observed that phosphorylation of Akt in omentin-1 treated CVSMCs was blocked by Compound C, indicating that Akt activation is partially AMPK-dependent while inhibition of Akt had no effect on omentin-1 induced activation of AMPK. Together, these results demonstrate that omentin-1 alleviates arterial calcification by activating AMPK-dependent Akt signaling. However, it remains unclear how AMPK regulates Akt activation in this cascade under omentin-1 treatment.

In conclusion, we demonstrated that exogenous omentin-1 attenuates osteoblastic differentiation of VSMCs through AMPK/Akt signaling pathway, thereby resulting in alleviation of arterial calcification. Moreover, we provided the first evidence that deletion of omentin-1 promotes arterial calcification in 5/6 NTP-induced mice. Taken together, these data suggest that omentin-1 represents a novel molecular mechanism mediating adipose-cardiovascular crosstalk and thus may serve as a novel potential biomarker and therapeutic target for vascular calcification and cardiovascular disease.

## MATERIALS AND METHODS

### Ethics statement

This investigation conforms to the Guide for the Care and Use of Laboratory Animals, NIH publication, 8^th^ edition, 2011. All the animal were formally approved by the Ethics Committee of the Second Xiang-Ya Hospital, Central South University.

### Reagents

Recombinant omentin-1 protein was purchased from R&D Systems (Minneapolis, MI, USA). Antibodies for AMPKα, p-AMPKα (Thr172), Akt, p-Akt (Ser473), ACCα, p-ACCα were purchased from Santa Cruz Biotechnology Inc (Waltham, MA, USA). Runx2 or omentin antibodies were purchased from Abcam (Cambridge, UK). Anti-mouse and anti-rabbit monoclonal IgG peroxidase conjugate antibody, anti-β-actin polyclonal antibody were purchased from Proteintech (Rosemont, USA). AMPK inhibitor, Compound C, and inhibitor of Akt, LY 294002, were purchased from Calbiochem (San Diego, CA, USA). AMPK and Akt specific siRNA oligonucleotides as well as the control siRNA oligonucleotides were purchased from Ribobio (Guangzhou, China). The ALP assay kit was purchased from Nanjing Jiancheng Bioengineering Institute (Nanjing, China). The fetal bovine serum, penicillin, and ptreptomycin were purchased from Gibco-BRL Co., Ltd (Grand Island, NY, USA). Alizarin Red S, pbs and calcitriol were purchased from Sigma Chemical Co., Ltd (St.Louis, MO, USA).

### Cell culture and *in vitro* calcification

Human VSMCs and mouse calcifying vascular smooth muscle cells (CVSMCs) were acquired and identified by a previously established method [35, 36]. VSMCs were cultured in Dulbecco's Modified Eagle's medium containing 10% fetal bovine serum at 37 °C under a humidified atmosphere containing 5% CO_2,_ and VSMCs induced by glycerophosphate were cultured in the above-mentioned medium including 10 mM β-GP. CVSMCs were seeded in Dulbecco's Modified Eagle's medium containing 4.5 g/L of glucose, 10% fetal bovine serum and 10 mM sodium pyruvate, as previously described [5]. Primary cells passaged for 3 to 6 times were used in the following experiments.

### Analysis of the ALP activity and osteocalcin secretion

Cells were cultured in the absence or presence of 100−400 ng/mL omentin-1, and washed three times with PBS. The cell layers were scraped into a solution containing 20 mM Tris–HCl, pH 8.0, and 150 mM NaCl, 1% Triton X-100, 0.02% NaN3 and 1 mM PMSF. After the lysates were homogenized by sonication for 20 seconds, the ALP activity was measured using an ALP kit using the spectrophotometric measurement of p-nitrophenol release at 37 °C. The ALP activity was normalized to the total protein content of the cell lysate.

OC secreted into the culture media was determined using a radioimmunoassay kit (DiaSorin, Stillwater, MN, USA). Bradford protein assay was used to normalize protein expression to the total cellular protein.

### Measurement of mineralized matrix formation

For Alizarin Red S staining, cells in 24-well plates were cultured in medium, with or without 400 ng/mL omentin-1 or vehicle for the indicated time. Then, the extent of the mineralised matrix was determined by Alizarin Red S staining [37]. Briefly, cells were fixed in 70% ethanol for 1 hour at room temperature and stained with 40 mM Alizarin Red S for 10 minutes. Next, cell preparations were washed with PBS to eliminate nonspecific staining. The stained matrix was photographed by a digital microscope.

For the measurement of calcium deposition, cells were washed in PBS and decalcified with 0.6 N HCl for 24 hours. O-cresolphthalein, was used to react with calcium and calcium content of the cell layer was normalised to the total protein content using the Bradford protein assay.

### Western blot analysis

Total protein extracts of cultured cell were prepared with RIPA lysate (Beyotime, China) and measured by BCA Protein Assay kit (Beyotime, Shanghai, China). 30ug of total protein were submitted to 12% SDS-PAGE and transferred onto 0.2 μm PVDF membranes (Pall, USA). The membranes were blocked by 5% milk or BSA as required, followed by incubation overnight with primary antibody, and then subjected to HRP-labeled secondary antibody for 1 hour at room temperature. The reaction was visualized with chemiluminescent assay.

### Inmunohistochemistry

Firstly, the five-micron-thick paraffin sections were pre-warmed to 60 °C for an hour in the oven and then were dewaxed in xylenes and rehydrated in grade ethanol baths. Secondly, antigen retrieval was carried out using boiling citrate buffer bath (pH=6) for 10 minutes and then artery sections were incubated in 3% hydrogen peroxide for 10 minutes at room temperature to block endogenous peroxidases. The blocking procedure was performed by using 5% fetal serum for an hour. Finally, sections were incubated by indicated primary antibody or PBS control overnight at 4 °C. The next day, sections were incubated with diluted secondary antibody for 30 minutes at room temperature. Positive immunoreactivity was detected using the kit and analysed by Image-pro Plus 6.0 software. All the experiments were repeated more than three times.

### Animal studies

To induce artery calcification in C57BL/6 mice, 5/6 nephrectomy with high-phosphate diet (5/6 NTP) were performed as previously described [30]. Briefly, six-week-old male C57BL/6 WT mice were randomly divided into two groups: a sham operation group and a 5/6 NTP operation group. The mice were anesthetized by isoflurane general anaesthesia (1–1.5% in medical oxygen) on a homeothermic blanket at 37°C before operation. Suitable anaesthesia of mice was confirmed frequently by the absence of the pedal withdrawal reflex. Along with the operation procedures, heart rate and respiration rate were continuously monitored by a specific instrument (MouseOx, Starr. USA). Subsequently, 5/6 nephrectomy of one side mice renal was performed with adrenal gland reserved, and another side of renal was totally removed two weeks later. Then, all the mice received a high-phosphate diet (0.9% Pi) after completing renal ablation for the duration of the study to accelerate artery calcification. Uraemia induced arterial-calcification mice model was established successfully after 5/6 NTP for twelve weeks as reported previously.

In some specific group, the adenovirus-encoding omentin-1 (Ad-Ome) or control adenovirus-encoding β-galactosidase (Ad-Gal) (1×10^8^ plaque-forming units [p.f.u.]) were injected into the tail vein of mice one time per week for four weeks. If needed, LY294002 (20 mg/kg, dissolved in dimethyl sulfoxide, i.v.) or Compound C (20 mg/kg, dissolved in dimethyl sulfoxide, i.v.) was injected into 5/6 NTP mice one day before the first injection of Ad-Ome and every other day for four weeks.

Omentin-1-deficient (omentin-1^-/-^) mice were kindly provided by Prof. Hui Xie and were fed in a method as described previously [38]. Six-week-old male omentin-1^-/-^ mice (n=12) and their WT male littermates (n=12) were used in the following experiment and were subjected to 5/6 nephrectomy or sham operation. Mice were euthanized with cervical dislocation, and then vesical adipose tissues, aortas and blood samples were harvested.

Artery calcification of mice was detected by Alizarin Red S (0.5%, pH 9.0, Sigma) staining and Von Kossa staining and the ALP activity was measured in the same way as in previous article [32, 39]. The calcium content was measured using O-cresolphthalein Complexone method, as previously described. [6] Briefly, dried artery samples were decalcified with HCl. The calcium content in HCL supernatants was determined using the O-cresolphthalein method. Total protein was quantified using the Bradford protein assay. The calcium content was normalized with protein content and expressed as microgram calcium per milligram protein. The plasma mouse omentin-1 was determined by mouse omentin-1 ELISA kit (Uscn Life Science Inc, Wuhan, China). Moreover, the expression of specific proteins in the thoracic aorta was identified by western blot and immunohistochemistry.

### Statistical analysis

SPSS 23.0 for Windows (SPSS, Chicago, IL, USA) was used for all statistics. Data are presented as means ± SD unless specially stated. Pair or unpaired Student's t-test was performed when comparing two groups. Comparisons were made using a one-way or two-way ANOVA with the Tukey’s HSD post hoc analysis for multiple comparisons, and differences at p< 0.05 were considered statistically significant.

## Supplementary Material

Supplementary Figures

Supplementary Table 1
