# Supplementary material for: Adipose tissue-derived omentin-1 attenuates arterial calcification via AMPK/Akt signaling pathway
Source: Aging (Albany NY). 2019 Oct 25;11(20):8760–76. doi: 10.18632/aging.102251 (PMC6834406; doi:10.18632/aging.102251)
Supplement: Supplementary Figures [file aging-11-102251-s002.pdf]

SUPPLEMENTARY FIGURES

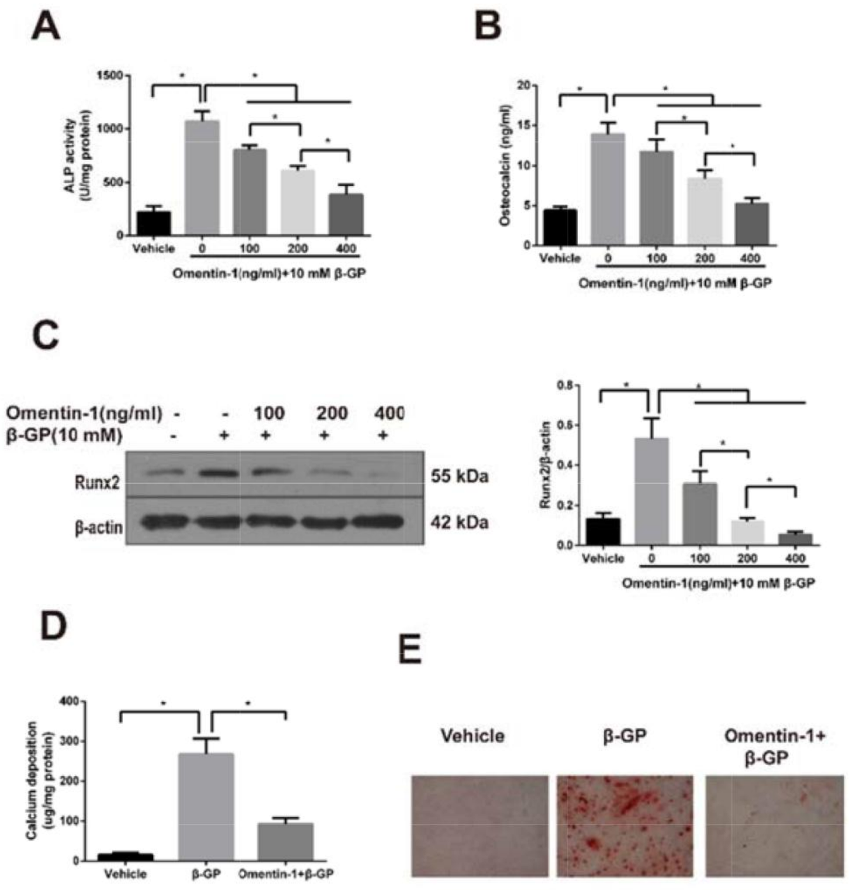

Supplementary Figure 1. Effects of omentin-1 on osteoblastic differentiation of HAVSMCs cultured with β-glycerophosphate (β-GP). (**A**) Effects of omentin-1 on the ALP activity in β-GP treated primary human arterial VSMCs (HAVSMCs). The cells were cultured with or without omentin-1 (100-400 ng/mL) for the indicated time points. ALP activity was measured by an ALP kit, normalized to the cellular protein contents. (**B**) Effects of omentin-1 on osteocalcin (OC) secretion in β-GP treated VSMCs. The cells were treated with or without omentin-1 (100–400 ng/mL) for 48 hours, and then OC secretion of the cells was determined by radioimmunoassay, normalized to the cellular protein contents. (**C**) Effect of omentin-1 on Runx2 expression in β-GP treated VSMCs. The cells were cultured for 48 hours with or without 100-400 ng/mL omentin-1. The data were presented as densitometric ratios of Runx2/β –actin. (**D**) Effects of omentin-1 on calcium deposition. The cells were cultured for 21 days with β-GP or β-GP+omentin-1. Representative results were shown. (**E**) Alizarin Red S staining view of either β-GP or β-GP+omentin-1-treated VSMCs for 21 days. Representative microscopic pictures were shown. Results are represented by mean ± SD with 3 replicates for each group. (*$p < 0.05$). One-way ANOVA with the Tukey's HSD post hoc analysis was adopted.

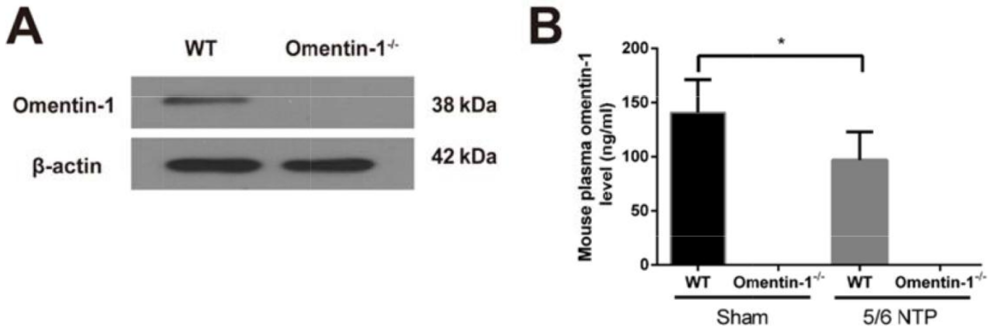

Supplementary Figure 2. Expression of omentin-1 in omentin-1 deletion mice and in chronic kidney disease state of 5/6 NTP-induced mice. (**A**) Expression of omentin-1 in vesical adipose tissues of omentin-1 null mice. (**B**) Plasma mouse omentin-1 concentration in omentin-1-/- mice and their littermates twelve weeks after sham operation or 5/6 nephrectomy (*$p < 0.05$ vs. sham control, n=6/per group, unpaired Student's t-test).
