# Supplementary material for: Adipose tissue-derived omentin-1 attenuates arterial calcification via AMPK/Akt signaling pathway
Source: Aging (Albany NY). 2019 Oct 25;11(20):8760–76. doi: 10.18632/aging.102251 (PMC6834406; doi:10.18632/aging.102251)
Supplement: Supplementary Table 1 [file aging-11-102251-s001.pdf]

SUPPLEMENTARY TABLE

Supplementary Table 1. Circulating levels of omentin-1 in mice with different treatments.

Groups	Sham		5/6NTP			
	Ad-Gal	Ad-Ome	Ad-Gal	Ad-Ome	Ad-Ome+ LY294002	Ad-Ome+ Compound C
Human omentin-1 (ng/ml)	0	163±19	0	168±12	164±15	162±14
Mouse omentin-1 (ng/ml)	137±13	130±18	107±15	112±16	108±18	113±19

Data are mean ± SD.
